# Variable Transcription Factor Binding: A Mechanism of Evolutionary Change

**DOI:** 10.1371/journal.pbio.1000342

**Published:** 2010-03-23

**Authors:** Patricia J. Wittkopp

**Affiliations:** 1 Department of Ecology and Evolutionary Biology, University of Michigan, Ann Arbor, Michigan, United States of America; 2 Department of Molecular, Cellular, and Developmental Biology, University of Michigan, Ann Arbor, Michigan, United States of America

## Abstract

This primer discusses a study from Bradley et al. in which changes in transcription factor binding between two *Drosophila* species are revealed by chromatin immunoprecipitation and high-throughput sequencing.

How an organism uses its genome to construct a complex body with many different types of cells, tissues, and organs remains a central question in biology. The key to creating diverse structures from a common set of genomic instructions is knowing not only which elements are relevant to a particular task but also when and where they must be used to assemble the tools needed to build the final form. For a cell, gene products (i.e., RNAs and proteins), which are produced from coding DNA, are the primary tools, whereas *cis*-regulatory elements, which are typically composed of non-coding DNA located near the coding sequence they regulate, determine how much (if any at all) of a gene product is made in a particular cell.

During the last decade, researchers have discovered that the collection of proteins found in different animals is remarkably similar. In fact, many proteins are found not only in animals, but also in fungi and plants; some are even shared with bacteria. This unexpected—and truly astounding—finding has changed scientists' thinking about how biological diversity evolved; while it was once believed that changes in the sequences of proteins were primarily responsible for phenotypic differences within and among species, it is now clear that changes affecting the production (i.e., “expression”) of proteins also play a prominent role [1]. This evolutionary potential of gene regulation was recognized long before the molecular mechanisms controlling this process were understood [2],[3], but only recently have researchers begun to identify regulatory changes that contribute to the evolution of specific traits (e.g., [4]–[8]).

## Transcription Factors and Their Binding Sites Mediate Expression Divergence

A key step controlling protein expression is the biochemical interaction of a class of proteins—transcription factors—with *cis*-regulatory DNA sequences. These interactions are mediated by the DNA binding domain of the transcription factor and the nucleotides within the c*is*-regulatory DNA to which it binds. Most *cis*-regulatory sequences are bound by more than one type of transcription factor, and most transcription factors bind to the *cis*-regulatory sequences from several genes. The recruitment of different combinations of transcription factors to different genes allows expression of each gene to be regulated independently. Mutations that alter the activity or availability of transcription factors, as well as mutations that alter the *cis*-regulatory sequences to which they bind, can change gene expression. Both types of changes contribute to evolution; however, studies from a variety of organisms suggest that mutations affecting *cis*-regulatory activity are the predominant source of expression divergence between species (e.g., [9]–[12]). Because gene expression is an essential component of how cells work, and because changes in gene expression often alter phenotypes, mutations that affect gene expression can affect fitness and contribute to adaptive evolution.

Despite their importance, identifying specific changes in DNA sequence that alter *cis*-regulatory activity, and understanding how these changes affect gene expression, has been challenging. For example, finding non-coding sequences that harbor *cis*-regulatory information for a gene often requires extensive experimental investigation. Comparing sequences from different species can accelerate this work because regulatory sequences are typically more conserved than non-functional DNA [13], but ultimately, experimental validation is still required. Once a *cis*-regulatory sequence is in hand, the subsets of that sequence that bind to individual transcription factors must be identified. This process is made even more challenging by the fact that researchers rarely know a priori which transcription factors' binding sites they seek. Finally, even when all transcription factor binding sites within a *cis*-regulatory sequence are known, it is generally impossible to predict precisely how changes in these sequences will affect transcription factor binding and ultimately gene expression. Such roadblocks are slowly being cleared. Furthermore, techniques such as “ChIP-seq” [14] and “ChIP-chip” [15] are developed that provide researchers with alternative ways to study the relationships between sequence variation, transcription factor binding, and gene expression.

## Tracking Changes in Transcription Factor Binding across the Genome

**Figure 1:**
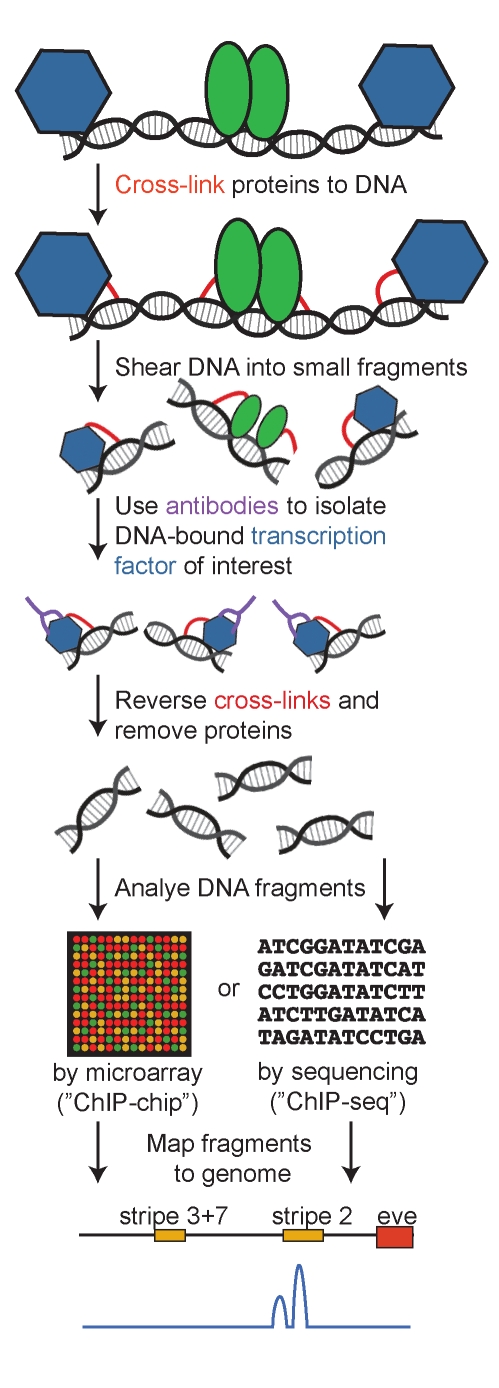
ChIP-chip and ChIP-seq reveal transcription factor binding genome-wide. DNA fragments bound by a transcription factor of interest (represented by blue hexagons) are recovered as shown. After mapping each DNA fragment to the genome, the relative frequency of each base within the recovered pool of DNA is summarized as a histogram. An example of such a histogram is shown in blue at the bottom of the figure for the 5′ region of the *Drosophila even-skipped* (*eve*) gene containing the enhancers that drive expression in embryonic stripes 2, 3, and 7. The shape of this histogram mimics ChIP-seq data for binding of the Giant transcription factor in *D. melanogaster* and *D. yakuba*, as shown in Figure 1A of [19].

“ChIP-seq”, which stands for chromatin immunoprecipitation followed by sequencing, allows binding sites for a given transcription factor to be located genome-wide and estimates the relative affinity of the transcription factor for each sequence. Its predecessor, “ChIP-chip”, which stands for chromatin immunoprecipitation followed by microarray (or “chip”) analysis, performs a similar function. In both cases (Figure 1), cells are treated with a chemical that links proteins bound to DNA in place and then the DNA/protein complexes (i.e., “chromatin”) are extracted and sheared into tiny pieces, each of which contains only a few hundred base-pairs of DNA. An antibody that specifically recognizes the transcription factor of interest is then used to isolate (i.e., “immunoprecipitate”) the fragments of DNA that were bound to that transcription factor. Finally, the chemical links between proteins and DNA are reversed, allowing the naked DNA to be recovered. These naked DNA fragments are either sequenced (ChIP-seq) or hybridized to a microarray (ChIP-chip) to determine their identity and mapped onto an existing genome sequence. Sequences observed most often are inferred to have the highest affinity for the transcription factor.

Insight into regulatory evolution can be obtained using ChIP-chip or ChIP-seq by comparing the binding sites for the same transcription factor in genomes from multiple species. Such comparisons have thus far been made for three transcription factors in multiple species of yeast [16],[17], for four transcription factors in mice and humans [11],[18], and, as reported by Bradley et al. [19] in this issue of *PLoS Biology*, for six transcription factors in two closely related species of fruit flies (*Drosophila*). In yeast, less than half of the binding sites identified for each transcription factor were observed in any pair of species (i.e., 22–34% in [16] and 7–42% in [17]). A similar pattern was observed between the mouse and human genomes: 11–59% of binding sites identified in one species were also observed in the other, depending on which transcription factor was examined [11],[18]. In flies, however, nearly all binding sites (i.e., 95–99%) detected for a given transcription factor were found in both species. This greater consistency of binding sites between *Drosophila* species results primarily from the more recent divergence of the species examined, but may also reflect increased sensitivity of ChIP-seq compared to ChIP-chip and/or the specific transcription factors chosen to analyze. Examining divergence of transcription factor binding over different evolutionary timescales provides a more complete understanding of the evolutionary process than any single comparison alone: closely related species allow changes in DNA sequence to be associated with changes in gene expression, while more distantly related species can reveal regulatory changes associated with major phenotypic transitions.

## Evolutionary Changes in Transcription Factor Binding Site Affinity

Bradley et al. [19] consistently detected binding of the same transcription factors to regions of DNA in *D. melanogaster* and *D. yakuba* that have a common evolutionary origin; however, the relative affinity of these binding sites often differed between species. This suggests that evolutionary changes in the DNA sequence of *cis*-regulatory regions have occurred that alter the strength of the interaction between transcription factors and their binding sites without completely eliminating binding. Such changes are possible because transcription factor binding sites are degenerate. That is, transcription factors typically bind to multiple—although usually similar—sequences, but do so with different affinities. Mutations affecting the affinity of a single transcription factor binding site can be sufficient to alter *cis*-regulatory activity, although this is not always the case (e.g., [20]).

Surprisingly, Bradley et al. [19] observed coordinated changes in binding affinity for all six transcription factors examined in some genomic regions. This pattern is not expected to result from random mutations of DNA sequences that alter affinity of individual binding sites. Rather, it suggests a different type of divergence that affects transcription factor binding. The most likely explanation for this divergence is that differences between species exist for how orthologous regions of DNA are packaged within cells. In order to fit into the nucleus of a cell, DNA is wrapped around histone proteins, which are assembled into nucleosomes. The precise form of this DNA/protein packaging (i.e., “chromatin”) varies among regions of the genome and affects how or if transcription factors can bind to DNA. Individual transcription factor binding sites are usually less than 10–base-pairs long, whereas each nucleosome encompasses 146 base-pairs of DNA, suggesting that even localized changes in chromatin structure can simultaneously affect the accessibility of multiple transcription factor binding sites. Consistent with this idea, Bradley et al. [19] found that the presence or absence of a DNA sequence thought to affect nucleosome positioning was correlated with coordinated changes in transcription factor binding between species.

## Evolutionary Noise or Adaptive Divergence?

With patterns of transcription factor binding now described for multiple species, we are faced with the challenge of understanding how differential transcription factor binding affects organisms. For example, which changes in transcription factor binding actually alter gene expression? One way to begin addressing this question is to compare the changes in transcription factor binding identified using ChIP-chip and ChIP-seq to species-specific patterns of gene expression. If changes in transcription factor binding tend to cause changes in gene expression, a correlation between the presence or affinity of transcription factor binding sites and expression of the genes they regulate may be observed. Perhaps even more important (and challenging) than answering this question is determining which changes in gene expression actually alter organismal fitness. Resolving this issue will help distinguish between interspecific expression differences that are likely to be adaptive and the product of natural selection and those that are likely to be neutral and the product of mutation and drift.

Despite these outstanding questions, researchers have begun speculating about the evolutionary forces driving expression divergence based on patterns of gene expression within and between species (reviewed in [21]). Such analyses generally conclude that natural selection has played some role in expression divergence, but the relative frequency of adaptive and neutral changes remains unclear. Bradley et al. [19] observed differences in transcription factor binding between species that were similar in regions of the genome thought to act as functional *cis*-regulatory elements and those thought to have no such activity. This finding could suggest that the majority of changes in transcription factor binding may have little to no effect on gene expression These data do not necessarily suggest, however, that all changes in transcription factor binding are neutral. It remains plausible (even likely) that a subset of changes in transcription factor binding observed by Bradley et al. [19] alter gene expression and may have contributed to adaptive evolution.

Undoubtedly, tremendous progress has been made in recent years towards understanding regulatory evolution and its role in adaptive evolution; however, key questions remain about the relationship of sequence variation to transcription factor binding, transcription factor binding to gene expression, gene expression to phenotypic variation, and phenotypic variation to fitness in the wild. Fortunately, technological advances and new genomic tools, such as those described herein, are opening new avenues for systematically exploring these relationships.
